# Corrosion Inhibition of Mild Steel by Coumarate‐Based Ionic Liquids and Coatings

**DOI:** 10.1002/cphc.202400477

**Published:** 2024-09-16

**Authors:** Sharon Monaci, Daniele Mantione, David Mecerreyes, Maria Forsyth, Anthony Somers

**Affiliations:** ^1^ Institute for Frontier Materials Deakin University Burwood VIC 3125 Australia; ^2^ POLYMAT University of the Basque Country UPV/EHU 20018 Donostia-San Sebastian Spain; ^3^ IKERBASQUE Basque Foundation for Science 48009 Bilbao Spain

**Keywords:** Corrosion, Ionic Liquids, Inhibitors, Coatings

## Abstract

The use of ionic liquids (ILs) as corrosion inhibitors is gaining significant attention due to their attractive properties such as high inhibition efficiency and ability to absorb onto metal surfaces. In this work, six protic ILs, based on the coumarate anion in combination with the nitrogen containing ammonium, pyrrolidinium and imidazolium cations with a short or long alkyl chain attached to the nitrogen atom, have been synthesized and evaluated as corrosion inhibitors for mild steel. The anticorrosion properties of these ILs in solution as inhibitors were investigated electrochemically and the metal surface was analyzed by Scanning Electron Microscopy. Moreover, the IL prepared from the coumarate anion and N‐dimethyl‐N‐tetradecyl ammonium ([DTA]Cou) was incorporated into an acrylic UV‐cured coating formulation as an additive and by designing a similar ionic monomer which covalently links to the acrylic coating formulation. Both coatings were analyzed using impedance spectroscopy during 11 days of exposure to a solution of 0,01 M NaCl, confirming the high performance of the inhibitor in both solution and when incorporated into a coating. The synthesized ILs present efficiencies in solution exceeding 70 %, in particular the ILs [DTA]Cou and the tetradecyl imidazolium coumarate ([C14Im]Cou) showed efficiencies of 88 % and 91 % respectively. Surface analysis after 24 h confirmed that the inhibitors efficiently adsorb onto the mild steel surface to form a protective film. The obtained inhibitors showed interesting anticorrosion behaviors and demonstrated how different cations and an increase in the chain length affect the corrosion inhibition properties.

## Graphical abstract

The combination of a coumarate anion with other organic corrosion inhibitors into Ionic Liquids demonstrated effective corrosion protection in solution with an inhibition efficiency up to 92 % and showed how an increase in the alkyl chain length affects the inhibition performance. Moreover, the integration of one inhibitor into a coating through two different strategies proved the efficiency of the inhibitor when incorporated into a polymerizable matrix.

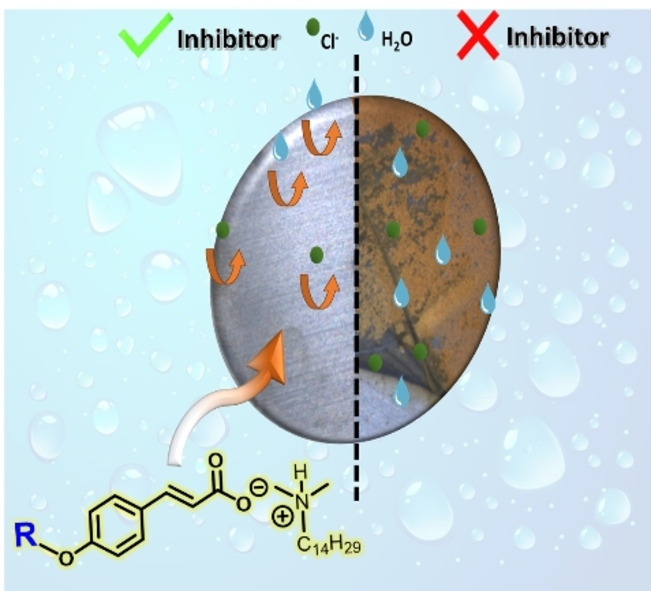

## Introduction

Corrosion represents a persistent global challenge, leading to substantial economic losses and safety concerns.^[1]^

One of the many areas impacted by corrosion is the construction of metallic structures exposed to atmospheric conditions (i. e. framework of buildings, machine tools), where iron is used as the metal of choice thanks to its low cost and high strength^[2]^ Unfortunately, the poor corrosion resistance of iron raises concerns about the premature failure of such structures, posing economic and safety risks.

Therefore, the use of different strategies to prevent and inhibit corrosion is crucial. Among the various approaches, corrosion inhibitors are one of the most used as they can easily be applied to pre‐existing structures through coatings. Previously, the majority of inhibitors were inorganic and based on metallic compounds such as chromate, phosphate and molybdate. However, the banning of the classic corrosion inhibitor, Cr VI, due to its toxicity,^[3]^ has created the need to explore safer alternatives that can also guarantee excellent performance. For these reasons, organic compounds have emerged as promising alternatives to inorganic‐based corrosion inhibitors. Usually, organic inhibitors should contain heteroatoms and pi‐electrons that allow the molecule to interact with the free d‐orbital of the metal, forming a layer onto the surface by physical and chemical absorption[^4^, ^5^, ^6^] and protecting it from water and the attack of aggressive species. Among organic inhibitors, amino acids, alcohol and amines like imidazole and pyrrolidine have demonstrated an effectiveness in reducing the corrosion process of metals[^7^, ^8^, ^9^] Several studies emphasize the importance of alkyl chain length on the corrosion process[^7^, ^10^, ^11^] indicating that the corrosion inhibition efficiency increases with the chain length.

More recently, organic salts, particularly Ionic Liquids (ILs), are being investigated as corrosion inhibitors due to their ionic character which favors the interaction with the metal surface, high stability, and high efficiency in different aggressive media.^[12]^

Typically, ILs are selected based on the cation, often combined with inactive anions like bromide or chloride. However, recent studies presented organic salts where both cation and anion are active in inhibiting corrosion therefore resulting in properties that can result in a synergic effect between the cation and the anion.[^6^, ^9^, ^13^, ^14^]

Among various organic anions promoting metal surface interactions, cinnamate derivatives incorporated with rare earth elements have gained increasing interest[^15^, ^16^] as promising alternatives to chromate‐based inhibitors.

Previous studies[^16^, ^17^, ^18^] demonstrated a synergistic effect between the rare earth cation (Cerium or Lanthanum) that generally act as cathodic inhibitors and a cinnamate derivative, coumarate, which is mainly anodic, resulting in the new salts acting as mixed inhibitors. Furthermore, these ionic liquids and salts can be added to polymer coatings that provide an additional physical barrier against corrosion. In the case of cinnamate‐based ILs, it has been demonstrated that metal samples coated with an epoxy resin doped with the IL lanthanum 4 hydroxy cinnamate show less corrosion and absence of pitting with respect to the epoxy only coating.^[19]^

However, given the limited availability and potential high cost^[20].^of rare earth compounds, it is essential to find alternative cations offering similar properties but greater availability.

Among the various cinnamate derivative ILs, coumarate anions have demonstrated high efficiency as inhibitors, not only when incorporated with REM, but also with other cations such as triethylamine and vinyl imidazolium[^21^, ^22^] These ILs have also been effectively incorporated into a coating by linking one of the ions to the polymer matrix.^[22]^

This work presents an in‐depth investigation into the combination of the coumarate anion with a variety of nitrogen‐containing cations including long and short alkyl chains. The cations chosen for this study are already known for their anticorrosion properties[^9^, ^10^, ^14^] but have never been explored in combination in ILs with coumarate as the anion.

For this purpose, six novel ILs were synthesized and their corrosion inhibition behavior in 0.01 M NaCl solution on AS1020 mild steel^[23]^ (composition reported in Table 1, ^[14]^) was assessed through Potentiostatic Electrochemical Impedance Spectroscopy (PEIS) and Potentiodynamic Polarization (PP). Additionally, different strategies to incorporate the most effective IL into an acrylic UV‐coating were explored, and the resulting coatings were investigated to determine the influence of the inhibitor on the coating's barrier effect.

**Table 1:** Composition of commercial AS1020 mild steel.

C	Mn	Cr	Si	Al	Other	Fe
0.26 %	0.45 %	0.14 %	0.19 %	0.18 %	0.35 %	Balance

## Experimental

### Materials

All the purchased chemicals were used without any further purification. AS1020 mild steel and Milli‐Q water were used for the corrosion tests and preparation of solutions. The composition of AS1020 mild steel is shown in Table 1.^[14]^

Trans‐p‐coumaric acid was purchased from TCI (>98 wt %). Triethylamine (TEA) (Sigma Aldrich, >99.5 wt %), 1‐methylimidazole (MIM) (Sigma Aldrich, >99 wt %), 1‐methylpyrrolidine (MPyr) (Sigma Aldrich, 98 wt %) and N,N‐dimethyltetradecylamine (DTA) (Sigma Aldrich, >95 wt %) were the chosen commercial nitrogen‐based molecules. 1‐bromotetradecane (TCI, >97 wt %) was used to add a 14 carbon alkyl chains to these compounds. Methacryloyl chloride (Sigma Aldrich, >98 wt %, containing 200 ppm monomethyl ether hydroquinone as stabilizer) and methacrylic acid (Sigma Aldrich, >99 wt %, containing 250 ppm monomethyl ether hydroquinone as inhibitor) were used to add a polymerizable moiety. Dipropylene glycol diacrylate SR508 was purchased from Arkema Sartomer® Americas. 2‐Hydroxy‐2‐methylpropiophenone (Darocur) was purchased from Merck^®^.

### Synthetic Procedure

#### Synthesis of Coumarate Monomer MHCou

**Figure 1:**
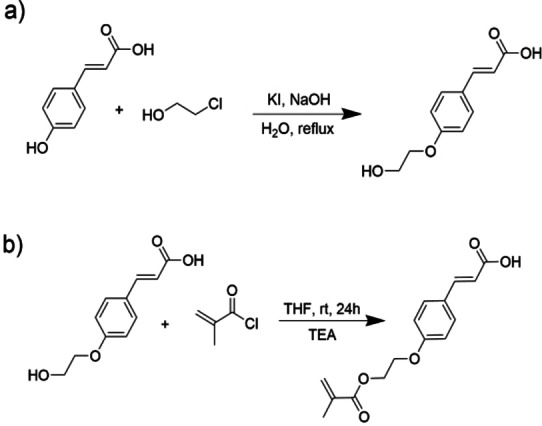
Synthetic steps for the synthesis of MHCou.

The synthesis of MHCou was conducted following the same procedure presented by Quites et al.^[24]^ As reported in step (a) of Figure 1, sodium hydroxide (3 eq) and potassium iodide (catalytic) were dissolved in water, then coumaric acid (1 eq) was added. The solution was stirred overnight at reflux until the color of the solution transitioned to dark red. The resulting solution was then washed with diethyl ether three times, and the product was isolated from the aqueous phase through precipitation by gradually adding hydrochloric acid. The collected solid was filtered using a Buckner funnel and dried under vacuum until a white powder was obtained.

In step (b) the product obtained from step (a) (1eq) was dissolved in a tetrahydrofuran solution of triethylamine (3 eq). The solution was then left stirring at room temperature for one day.

Subsequently, the solution was filtered, and the organic phase was separated by extraction with dichloromethane followed by three subsequent washes with acidic water. The organic phase was then dried with brine and sodium sulphate and the solvent evaporated under reduced pressure until a viscous yellow liquid was obtained. The product underwent purification via flash chromatography column using a hexane/ethyl acetate mixture in a 1 : 1 ratio. The final product achieved was a white powder. The NMR characterization matches with the one in literature^[24]^ 1H NMR (400 MHz, DMSO) δ 7.65–7.58 (m, 2H), 7.50 (d, J=16.0 Hz, 1H), 7.02–6.96 (m, 2H), 6.37 (d, J=16.0 Hz, 1H), 6.06–6.00 (m, 1H), 5.69 (p, J=1.6 Hz, 1H), 4.43 (dd, J=5.8, 3.3 Hz, 2H), 4.29 (dd, J=5.6, 3.4 Hz, 2H), 2.22–2.13 (m, 3H), 2.10 (s, 7H), 1.87 (t, J=1.4 Hz, 3H), 1.23 (m, 33H), 0.88–0.82 (t, 3H).

#### Synthesis of Tetradecylpyrrolidine (C_14_Pyr) and Tetradecylimidazole (C_14_Im)

**Figure 2:**
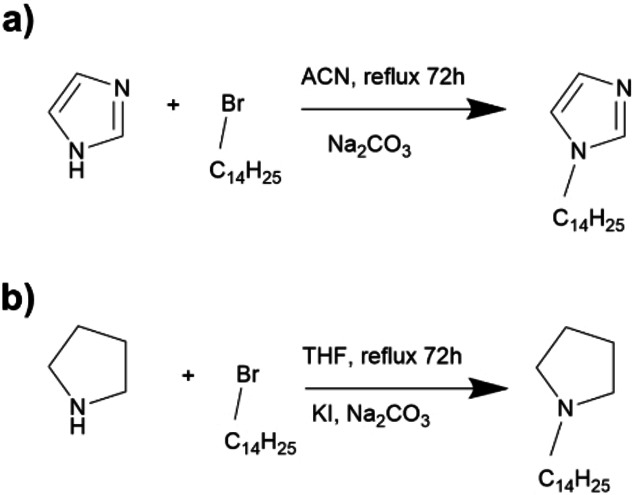
Synthesis of tetradecylimidazole **(a)** and tetradecylpyrrolidine **(b)**.

Following the scheme in Figure 2 **(a)**, which refers to the synthesis proposed by Sintra et al.^[25]^

Imidazole (1 eq) was dissolved in a solution containing sodium carbonate (3 eq) in ACN, then bromo tetradecane was added (2.3 eq) and the solution put at reflux. After 72 hours, the solvent was removed through reduced pressure and the product purified in a flash silica chromatographic column using a mixture of hexane/ethyl acetate 2/8 as eluent. The NMR characterization matched that in the literature^[25]^ and is reported in Figure S1. 1H NMR (400 MHz, DMSO) δ 7.62 (s, 1H), 7.14 (s, 1H), 6.88 (s, 1H), 3.93 (t, J=7.1 Hz, 2H), 1.67 (p, J=7.1 Hz, 2H), 1.22 (s, 22H), 0.84 (t, J=6.6 Hz, 3H).

Following the synthesis proposed by Saisai et al[26], pyrrolidine (1 eq, Figure 2 **(b)**) was dissolved in a solution containing potassium iodide (catalytic) and sodium carbonate (3 eq) in THF, then bromo tetradecane was added (2.3 eq) and the solution put at reflux. After 72 hours, the solvent was removed through reduced pressure and the product purified with a flash silica chromatographic column using a mixture of ethyl acetate/methanol 9/1 as eluent. The NMR characterization was a match for that in the literature[26] and is reported in Figure S2. 1H NMR (300 MHz, DMSO) δ 2.98 (s, 4H), 2.91–2.78 (t, 2H), 1.93–1.76 (m, 4H), 1.58 (m, J=9.5 Hz, 2H), 1.24 (s, 25H), 0.92–0.79 (t, 3H).

#### Synthesis of ILs for Solution Test

**Figure 3:**
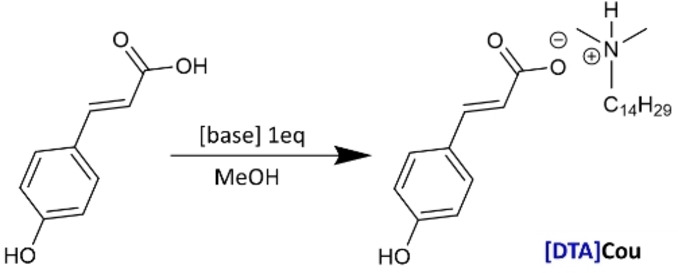
General procedure for the synthesis of ILs.

For the preparation of the protic ionic liquids 1 eq. of coumaric acid was added to a methanol solution and left stirring until complete dissolution, then an equimolar amount of the nitrogen‐based molecule was added to the mixture and the solution stirred overnight at 35 °C. The solvent was then removed under reduced pressure to obtain the corresponding products, all of them in form of viscous yellow liquids. The preparation of [DTA]Cou is reported in Figure 3, and the preparation of the other ILs was performed following the same procedure.

[DTA]Cou: 1H NMR (300 MHz, DMSO, Figure S3) δ 7.53–7.43 (m, 3H), 6.84–6.75 (m, 2H), 6.28 (d, J=15.9 Hz, 1H), 2.28–2.15 (m, 1H), 1.52–1.04 (m, 19H), 0.96–0.76 (m, 2H).

[MPyr]Cou: 1H NMR (300 MHz, DMSO, Figure S4) δ 7.50–7.36 (m, 3H), 6.80–6.74 (m, 2H), 6.27 (d, J=15.9 Hz, 1H), 2.62–2.51 (m, 3H), 2.33 (d, J=6.2 Hz, 3H), 1.79–1.64 (m, 3H).

[MIm]Cou: 1H NMR (300 MHz, DMSO, Figure S5) δ 7.57 (s, 1H), 7.50–7.42 (m, 3H), 7.30–7.20 (m, 1H), 7.09 (s, 1H), 6.87 (s, 1H), 6.81–6.77 (m, 1H), 3.17 (s, 3H).

[C_14_Im]Cou: 1H NMR (300 MHz, DMSO, Figure S6) δ 7.61 (d, J=2.4 Hz, 1H), 7.56–7.41 (m, 3H), 7.14 (s, 1H), 6.88 (s, 1H), 6.79 (d, J=8.3 Hz, 2H), 6.28 (d, J=15.9 Hz, 1H), 3.92 (t, J=7.1 Hz, 2H), 3.85–3.68 (m, 1H), 1.67 (p, J=7.1 Hz, 2H), 1.44–0.96 (m, 30H), 0.94–0.76 (m, 3H).

[C_14_Pyr]Cou: 1H NMR (300 MHz, DMSO, Figure S7) δ 7.54–7.43 (m, 4H), 6.79 (d, 2H), 6.28 (d, J=16.0 Hz, 1H), 3.24–3.11 (m, 3H), 3.09–3.00 (m, 1H), 2.11–2.00 (m, 1H), 1.96–1.86 (m, 2H), 1.66–1.53 (m, 2H), 1.34–1.17 (m, 22H), 0.84 (t, 3H).

#### Synthesis of [DTA]MHCou

**Figure 4:**
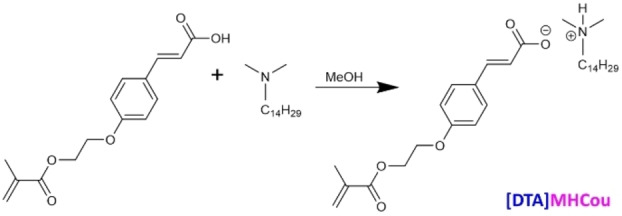
Synthesis of the IL [DTA]MHCou.

The preparation of the IL [DTA]MHCou is reported in **Figure** 4. The monomer MHCou was dissolved in methanol and stirred at room temperature, then an equimolar amount of N,N‐dimethyltetradecylamine was added and the solution was left stirring overnight. The solvent was then evaporated under reduced pressure and the NMR is reported in **Figure** **S8**. 1H NMR (300 MHz, DMSO) δ 7.66 – 7.47 (m, 3H), 6.99 (d, 2H), 6.38 (d, J=15.9 Hz, 1H), 4.56 – 4.37 (m, 3H), 4.37 – 4.27 (m, 2H), 1.53 – 1.34 (m, 7H), 1.34 – 1.12 (m, 28H), 0.85 (t, 3H). 13 C NMR (101 MHz, DMSO) δ 168.09, 159.84, 143.03, 130.21, 129.87, 127.38, 126.16, 117.62, 115.00, 65.95, 63.00, 59.80, 59.10, 45.10, 31.32, 29.04, 28.98, 28.73, 26.96, 26.87, 22.13, 20.80, 17.99, 14.12, 13.99. 13 C NMR (300 MHz, DMSO).

#### Electrochemical Characterization (PEIS and PP)

A BioLogic VMP300 ((Biologic, Seyssinet‐Pariset, France)) multichannel potentiostat controlled by EC‐Lab^©^ V11.44 software, was used for PEIS and PP experiments. The experimental setup utilized a standard three‐electrode cell configuration with a titanium mesh serving as the counter electrode (CE), AS1020 mild steel as the working electrode (WE), and an Ag/AgCl reference electrode (RE) placed in a Luggin capillary positioned close to the WE. The electrolyte used was an aqueous solution of Mili‐Q water containing 0.01 M NaCl and 8 mM of the inhibitor (for the analysis of inhibitors dissolved in solution), with 50 mL used for each test. For the electrochemical tests of coatings, the electrolyte used was an aqueous solution of Mili‐Q water containing 0.01 M NaCl. To minimize electrical interference, the cell was placed inside a Faraday cage during all measurements. For all electrochemical and immersion tests, triplicate samples were prepared for each condition to ensure reproducibility of the results.

The PEIS measurements were carried out at open circuit potential (OCP) over 24 h, using the OCP recorded immediately before each measurement. The impedance response was recorded after each hour in a frequency range of 10^5^ Hz to 10^−2^ Hz, using 10 points per decade and a sinusoidal perturbation amplitude of 10 mV. Representative OCP plots for the control and [TEA]Cou are shown in SI, Figure S16.

Following 24 hours of PEIS monitoring, the PP tests were performed. The curves were generated by varying the potential from −150 mV to +250 mV relative to the open circuit potential (OCP) at a scan rate of 0.167 mV/s. Before starting the PP scan, the OCV was stabilized for 30 minutes. The inhibitor efficiencies (IE(%)) were calculated through Tafel plot following Equation (1), using the software EC‐Lab^©^ V11.44.

The Tafel plots were extrapolated over a potential range of 10–25 mV on either side of the corrosion potential (*E_corr_*), where the linear segments of the anodic and cathodic branches of the PP curves intersect to define the corrosion current density (*i_corr_*).

(1) $$IE\left(\%\right)=\frac{i_{corr\;control}-i_{corr\left(i\right)}\;}{i_{corr\;control}\;}x100$$

Where *i_corr_* control is the current density measured when the sample is immersed in the control solution (0.01 M NaCl) and *i*_*corr(i)*_ is the current density measured when the sample is immersed in inhibited solution.

The equivalent circuit was fitted with the same software used for the PEIS and PP experiments, using a “Randomize + Simplex” fitting mode with the maximum number of iterations set at 6,000 iterations. For the fitting, the initial five high frequency points representing an arc were not included, as they can be considered an instrumental artifact related to the configuration of the three‐electrode cell.[^27^, ^28^]

Using the charge transfer resistance (R_CT_) extrapolated from the fitting the inhibition efficiency was also calculated at different time intervals (1 h, 12 h, 24 h) using Equation 2:^[29]^

(2) $${IE}_{fit}\left(\%\right)=\frac{R_{CT\left(inhibitor\right)}-R_{CT\left(control\right)}}{R_{CT\left(inhibitor\right)}\;}x100$$

Where R_CT(inhibitor)_ is the charge transfer resistance of the double layer at the interface steel/inhibitor and R_CT(control)_ is the charge transfer resistance of the double layer at the interface steel/control solution.

#### Sample Preparation for Corrosion Test in Solutions

The WE was a cylindrical rod of AS1020 mild steel with a diameter of 1 cm (surface area 0.79 cm^2^) encapsulated in epoxy resin to expose only one circular cut face of the cylinder to the solution. The WE was polished to 1200 grit, rinsed with distilled water, and stabilized for 24 h in a desiccator before immersion. Solutions of Mili‐Q water containing a concentration of 0.01 M NaCl and 8 mM of inhibitor were prepared by dissolving the respective amount of inhibitor in 1 L of solution and stirring overnight at room temperature. A control solution containing only 0.01 M NaCl was prepared in the same conditions.

#### Sample Preparation for Corrosion Test of Coatings

The WE consisted in AS1020 mild steel panels approximately 100×50x1 mm that were polished to 240 grit, rinsed with distilled water, and air‐dried before coating application.Following the application and UV curing of the coatings, a circular area with a diameter of 3 cm (surface area 7,1 cm^2^) was exposed to the Mili‐Q water solution containing 0.01 M NaCl.

#### UV‐Photopolymerization of Coatings

The acrylic formulation used as a control consisted of Dipropylene glycol diacrylate SR508 (Arkema) with Darocur23 (Speedcure 73) as the photoinitiator. The formulations containing the synthesized monomer were all prepared by addying 10 wt % of the monomer to 90 wt % Dipropylene glycol diacrylate SR508. After mixing, 5 wt % of Darocur23 (Speedcure 73) was added.

**Figure 5:**
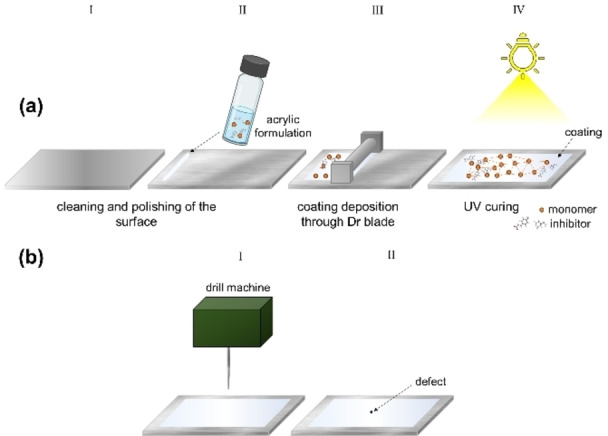
Preparation of the surface of AS1020 mild steel and curing of the coating **(a)**; introduction of a 1 mm diameter defect using a drill machine **(b)**.

As shown in Figure 5, the formulations were applied to AS1020 metal plates using a doctor blade technique to achieve a film thickness of 60 μm. These coatings were subsequently UV‐cured for 300 seconds with a 200 W UV lamp emitting at 360 nm. Post curing, the coated plates were placed in a desiccator for 24 hours to ensure complete drying. To investigate the impact of defects, a circular defect with a diameter of 1 mm was introduced into the coatings using a bench milling machine. FTIR of the formulations before and after UV curing are provided in Figure S9.

#### NMR Spectroscopy

^1^H Nuclear Magnetic Resonance (NMR) spectra were recorded at room temperature on a Bruker Avance DPX 300 at 300,16 MHz, using deuterated dimethyl sulfoxide (DMSO‐d6) as solvent.

#### FTIR spectroscopy

FTIR spectra were recorded on a Bruker Alpha‐P (Bruker, Billerica, MA, USA) from 500 to 3500 cm^−1^ with a resolution of 2 cm^−1^.

#### Scanning Electron Microscopy (SEM) and Energy‐Dispersive X‐ray Spectroscopy (EDS)

SEM micrographs of the sample surfaces were obtained following 24 hours of exposure to the inhibited solutions. A JSM‐IT300 LV SEM instrument, equipped with an Oxford X–Max 50 mm^2^ EDS detector operating at an accelerating voltage of 40 kV, was employed to capture surface images. The corresponding EDS spectra were collected using AZtec software (Oxford Instruments), providing elemental composition data to complement the surface morphology analysis.

#### Optical Microscope

AS1020 mild steel specimens were prepared following the same protocol used for the electrochemical characterization via PEIS and PP. After 24 h of immersion into the inhibited solutions, the surfaces of the samples were observed with a Leica MZ 7 optical microscope and the images were captured using the LAS V4.0 software.

## Results and Discussion

**Figure 6:**
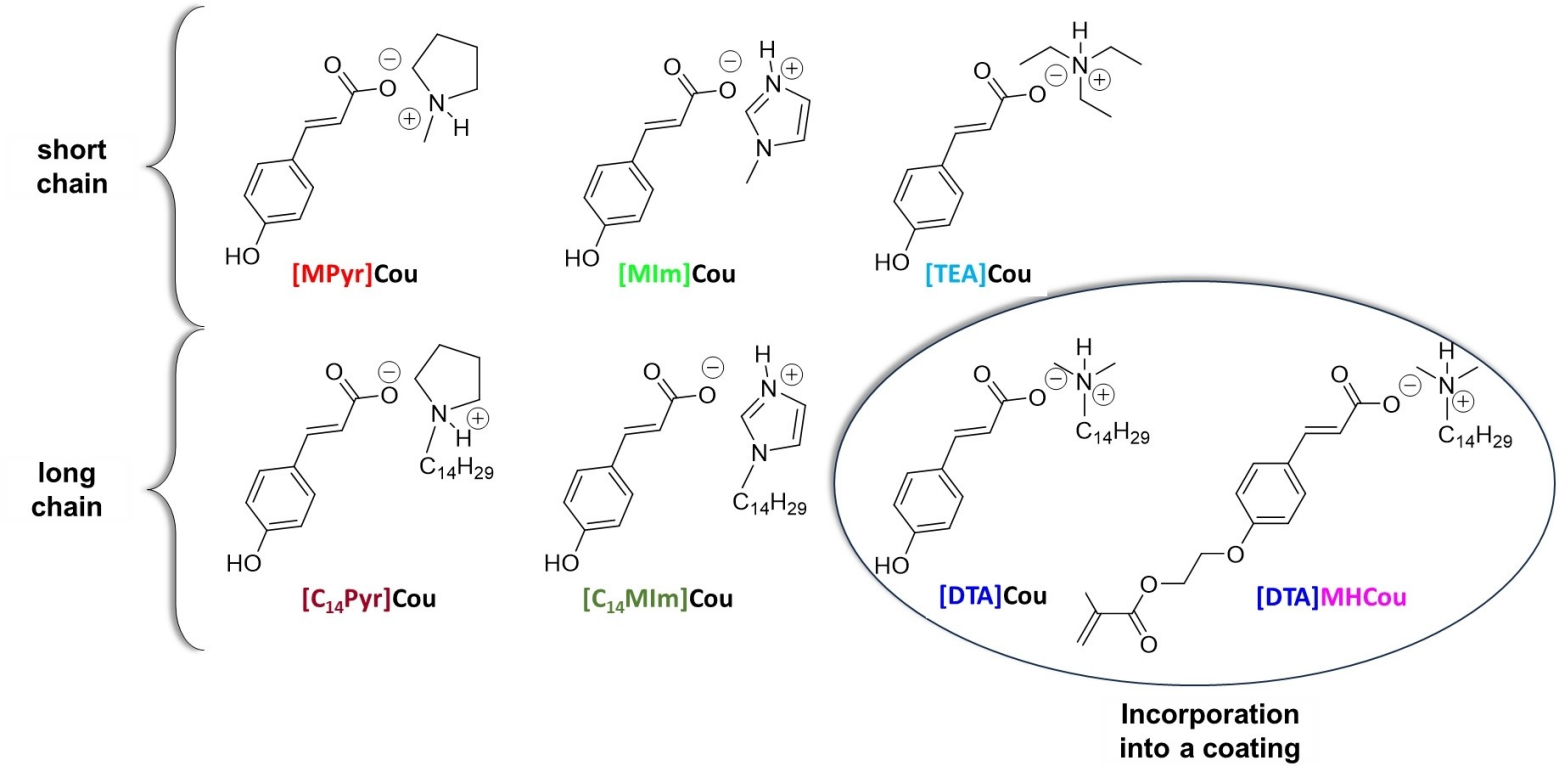
The different ILs investigated as corrosion inhibitors.

In this work, different coumarate‐based protic ionic liquids were synthesized and their structures and acronyms are reported in Figure 6. The three main cation structures are triethylammonium, pyrrolidonium, and imidazolium, with each one modified by adding a fourteen carbons chain. The ILs can be divided into short and long alkyl chain ones. Furthermore, an additional methacrylic coumarate IL was designed to be incorporated covalently into a methacrylic coating formulation.

### Surface Characterization

**Figure 7:**
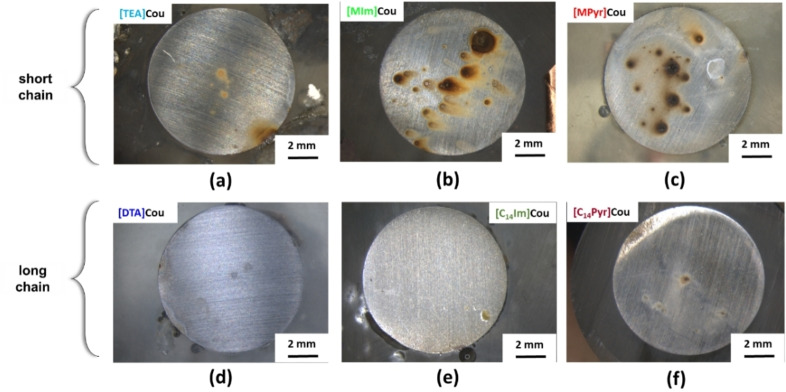
Microscope images of AS1020 mild steel immersed in 0.01 M NaCl solution containing 8 mM of respectively [TEA]Cou **(a)**, [MIm]Cou **(b)**, [MPyr]Cou **(c)**, [DTA]Cou **(d)**, [C_14_Im]Cou **(e)**, [C_14_Pyr]Cou **(f)**, after 24 h of immersion.

**Figure 8:**
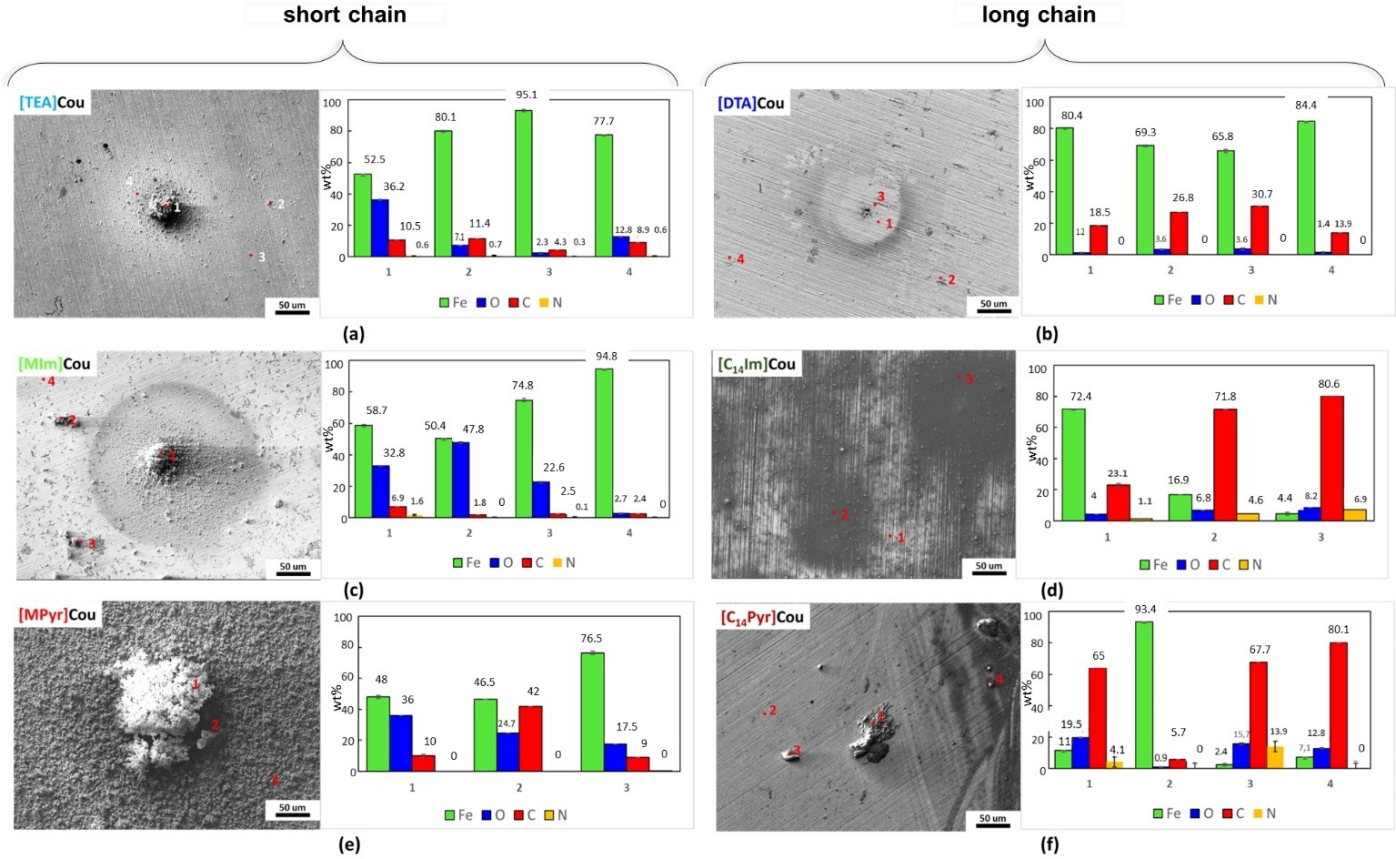
SEM micrographs of AS1020 mild steel after 24 h of immersion in 0.01 M NaCl solution containing 8 mM of [TEA]Cou **(a)**, [DTA]Cou **(b)**, [MIm]Cou **(c)**, [C_14_Im]Cou **(d)**, [MPyr]Cou **(e)**, [C_14_Pyr]Cou **(f)** with the respective EDS data.

The surfaces of the mild steel samples after 24 h of immersion in inhibited solutions were studied by optical microscopy (Figure 7) and SEM (Figure 8). Additionally, EDS analysis was conducted to identify and characterize the elements present on the sample surfaces post‐immersion. EDS data presented are representative of typical responses from deposits and general surface area for each sample. All the specimens immersed in the inhibited solutions present a less corroded surface than the one immersed in the control solution (Figure S10(a)). However, Figure 7 clearly illustrate that the ones immersed in the solutions of short alkyl chain ILs ([TEA]Cou **(a)**, [MIm]Cou **(b)**, and [MPyr]Cou **(c)**) present an appearance consisting with pitting corrosion, while for the specimens immersed in the inhibited solutions of [DTA]Cou **(d)**, [C_14_Im]Cou **(e)**, and . [C_14_Pyr]Cou **(f)** the surface after 24 h of immersion is mostly clean and does not present obvious signs of corrosion. In particular, while the surface of the samples immersed in the solutions containing [MPyr]Cou **(c)** and [MIm]Cou **(b)** present many pits surrounded by rust, the ones immersed in [C_14_Im]Cou **(e)** and [C_14_Pyr]Cou **(f)** presents an unaffected surface in the case of [C_14_Im]Cou and some signs of localized corrosion in the case of [C_14_Pyr]Cou. In all cases, the introduction of a long alkyl chain seems to mitigate the formation of localized corrosion and therefore increase the corrosion resistance.

The SEM micrographs and EDS analyses of the samples, as shown in Figure 8, generally focus on regions with heavy deposits, as the overall appearance of each sample is better reflected in the images taken with the optical microscope. Figure 8 shows that almost all the specimens present polishing marks and deposits of different sizes, while [MPyr]Cou (Figure 8<xfigr8**>(e)**) present substantial deposits, along with corrosion products that completely cover the surface.

EDS analysis reveals deposits characterized by higher concentrations of carbon and oxygen and a reduced percentage of iron than the surrounding areas. This, together with the comparison with the data obtained from the analysis of steel specimens before and after immersion in the control solution(Figure S10 (b), (c)), might suggest that the increase in oxygen can be related to the formation of oxidized form or iron (i. e. corrosion products), from corrosion or pit formation, while the increase in carbon content can be related to the presence of the inhibitor on the corrosion site.[^30^, ^31^] Moreover, an increase in the concentration of the nitrogen atom is observed for [C_14_Im]Cou (Figure 8 **(d)**) and [C_14_Pyr]Cou Figure 8<xfigr8**>(f)**), which can indicate the presence of the cation in the deposits.

As expected from the optical microscope images, the specimens immersed in the inhibited solutions of the long alkyl chain ILs present generally a higher percentage of carbon and sometimes nitrogen compared to the short alkyl chains, which on the other hand present a higher percentage of oxygen and iron. This can confirm the better ability of the long‐chain inhibitors to interact with the metal surface. In the case of [TEA]Cou Figure 8<xfigr8**>(a)** shows a large, isolated deposit surrounded by small precipitates, that present increased concentrations of carbon and oxygen (points 1,2, and 4) with respect to the control, suggesting the presence of both corrosion products and inhibitor, while the analysis on a point where the surface is clean and polishing signs are clearly visible present lower concentrations of carbon and oxygen. The samples exposed to [MIm]Cou (Figure 8<xfigr8**>(c)**) and [MPyr]Cou (Figure 8<xfigr8**>(e)**) present the highest percentages of oxygen among the tested inhibitors. In the case of the sample exposed to [MIm]Cou, while a specific area (point 1) suggests the presence of both corrosion products and inhibitor, analysis performed on other sites show that the carbon content remains within the range observed for the control, indicating minimal deposition of inhibitor residues. Furthermore, [MPyr]Cou (Figure 8<xfigr8**>(e)**) presents a high concentration of corrosion products covering all the regions analysed. From these preliminary results, it seems that two of the three short chain inhibitors, [MPyr]Cou and [MIm]Cou, present a high amount of corrosion products and therefore poor corrosion inhibition.

By comparison of [MPyr]Cou and [MIm]Cou with the respective long alkyl chain form [C_14_Pyr]Cou and [C_14_Im]Cou, it is clear that the introduction of a long alkyl chain improves the protection of the steel surface and reduce the formation of pits, coherently with the optical microscope images. The elemental composition of the respective surfaces in Figure 8 **(f) and (d)** changes drastically depending on the different points analyzed. Both surfaces exposed to [C_14_Pyr]Cou and [C_14_Im]Cou present a significant increase in the carbon content, followed by an increase of the nitrogen content in some areas.

Generally, the surfaces immersed in solutions containing the long alkyl chain inhibitors show the highest amounts of carbon and nitrogen, suggesting that these structures are favorable in attaching to the surface to form a protective film. Previous work with a cetrimonium coumarate[10] has shown that such long alkyl structures can form micelles in solution and act as surfactants that can interact with a corroding steel surface to protect it.

In particular, in the case of [C_14_Im]Cou in Figure 8 **(d)**, a higher coverage of the steel can be observed from the micrograph, which is not appreciable from the optical microscope (Figure 7 **(e)**), where the surface seems almost completely clean. Interestingly, it seems like the inhibitor is forming some aggregates on the surface of the steel. This was also shown in a similar study performed on two different imidazole‐bromide salts,^[32]^ where the long alkyl chain showed high stability and ability to cover the metal's surface.

Intrigued by the elevated carbon and nitrogen content indicated by the EDS analysis, an additional test was conducted by immersing a specimen in the [C14Im]Cou inhibited solution for 3 days. The results, shown in Figure S11, reveal significant inhibitor aggregate formation on the surface. These aggregates were substantial enough to be visible to the naked eye, suggesting that [C14Im]Cou facilitates the formation of large deposits over prolonged exposure. The aggregates were removed following the standard procedure for preparing, cleaning, and evaluating corrosion test specimens ASTM G1−03 (designation C 3.5 Table A1.1). After cleaning, the surface overall was still quite uniform but with some big pits appearing, meaning that [C_14_Im]Cou could lead to pitting corrosion over time. On the other hand, when the specimen was left into the [DTA]Cou solution for three days, the surface of the metal was mostly uncorroded (Figure S11**(c)**).

### Electrochemical Characterization of Coumarate‐Based ILs Dissolved in Solutions

#### Potentiostatic Electrochemical Impedance Spectroscopy (PEIS)

**Figure 9:**
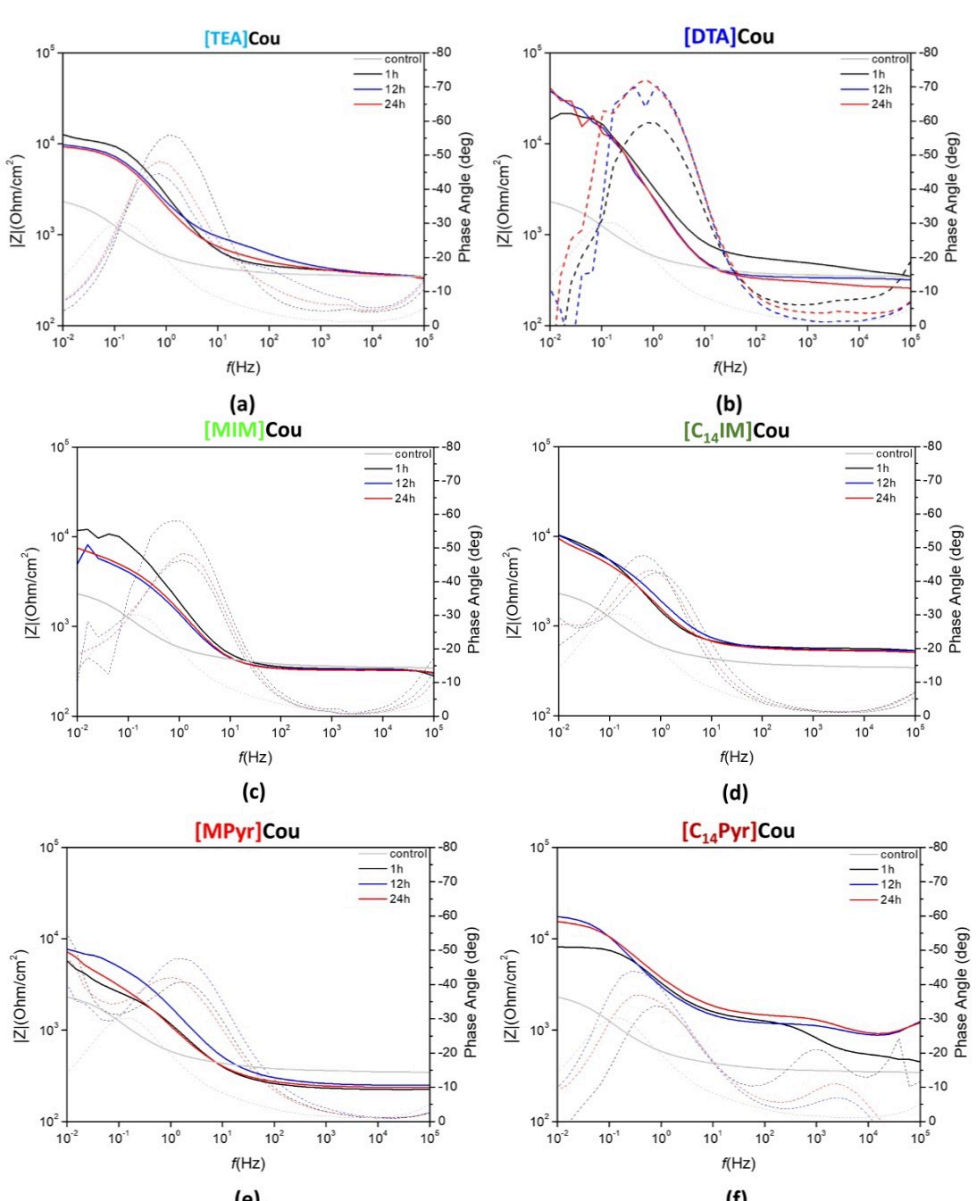
Impedance (solid lines) and phase angle plot (dotted lines) of AS1020 mild steel immersed in 0.01 M NaCl solution containing 8 mM of respectively [TEA]Cou **(a)**, [DTA]Cou **(b)**, [MIm]Cou**(c)**, [C_14_Im]Cou **(d)**, [MPyr]Cou **(e)**, [C_14_Pyr]Cou **(f)** after 1, 12 and 24 h of immersion compared to the control after 24 h (0.01 M NaCl, gray line).

The Bode plots of impedance and phase angle for AS1020 mild steel specimens immersed in solutions containing 0.01 M NaCl + inhibitor 8 mM after 1, 12 and 24 h of immersion are reported in Figure 9, while the Nyquist plots after 24 h of immersion are showed in Figure S12. The plots for the control (0.01 M NaCl) after 1, 12 and 24 h of immersion are reported in Figure S13, while in Figure 9 we only report the impedance of the control registered after 24 h of immersion (grey line).

The total impedance measured after 24 hours of immersion in the control solution was approximately 2450 Ohm/cm^2^ at 0.01 Hz, accompanied by a maximum phase angle magnitude of 30° at 0.17 Hz. Over the duration of immersion time from 1 hour to 24 hours, there was a noticeable shift in the time constant towards lower frequencies, likely attributable to the formation of corrosion products.

In contrast, solutions containing the dissolved inhibitors showed a general increase in both impedance and maximum phase angle magnitude, while the Nyquist plots clearly show that the size of the semicircles significantly increased with respect to the uninhibited system (control).The increase in impedance at low frequencies indicates a more capacitive behavior^[33]^ and is generally associated with the formation of a protective layer on the steel surface, which could result from either the accumulation of corrosion products or the adsorption of the inhibitor onto the surface.

In particular, the impedance spectra for [Tea]Cou **(a)**, [MPyr]Cou **(e)** and [MIm]Cou **(c)** exhibit lower values in the low frequencies range (10^−1–^–10^−2^ Hz) compared to their counterpart with long alkyl chains ([DTA]Cou Figure 9 **,(b)**, [C_14_Pyr]Cou Figure 9 **,(f)** and [C_14_Im]Cou Figure 9 **,(d))**. The long alkyl chain inhibitors also demonstrated higher and more stable impedances, confirming their stability during the immersion.

The inhibitor presenting the highest impedance after 24 h of immersion was [DTA]Cou, as seen in Figure S14, where the plots of impedance values at 0.01 Hz vs immersion time are reported. From the Bode plots, between 1 h and 12 h the impedance and phase angle stabilized around 40000 Ω and −70° respectively, with no appreciable shift observed in the phase angle and a small increase in the impedance between 12 h and 24 h, appreciable in Figure S14. This indicates that the inhibitor was able to effectively protect the steel surface over the entire duration of the analysis. Generally, all the inhibitors showed an important change in the impedance between 1 h and 12 h.

The time constant of [DTA]Cou showed a clear increase in magnitude from 50° to 70°, whereas [C_14_Pyr]Cou and [C_14_Im]Cou showed more subtle differences in peaks and angles. Notably, the phase angle plot of [C_14_Pyr]Cou presents an additional peak around 10^3^–10^4^ Hz, which is usually correlated to the presence of a film protecting the surface[34], in accordance with the literature, where similar structures have been investigated.^[9]^

In Figure S14, a comparison of the impedance values indicates that the [DTA]Cou inhibitor exhibits the highest average impedance, followed by [MIm]Cou and [TEA]Cou. This suggests that [DTA]Cou provides the most effective barrier against corrosion among the tested inhibitors.

**Figure 10:**
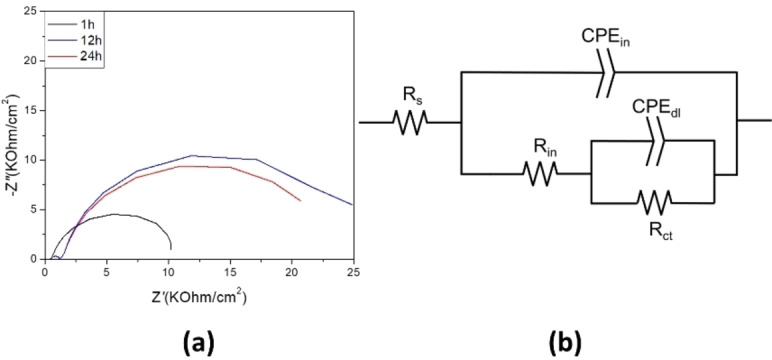
**(a)** Nyquist plots of of AS1020 mild steel immersed in 0.01 M NaCl +8 mM [DTA]Cou. **(b)** Equivalent circuit model used to fit PEIS data for AS1020 mild steel samples after 24 h immersion in 0.01 M NaCl + 8 mM [DTA]Cou.

An equivalent circuit model was used to describe the electrochemical processes happening during the immersion of the specimens in the inhibited solution of [DTA]Cou. Figure 10 reports the Nyquist plots after 1 h, 12 h and 24 h of immersion and the equivalent circuit model used for the fitting. The values of the different components can be found in Table S1 (control) and Table S2 ([DTA]Cou). The elements used in the equivalent circuits are: R_s_ the solution resistance; R_in_ and CPE_in_ the resistance and constant phase element (CPE) of the adsorbed inhibitor layer; R_ct_ and CPE_dl_ are respectively the charge transfer resistance and constant phase element of the double layer at the interface steel/inhibitor. A constant phase element (CPE) has been used instead of capacitance due to the inhomogeneous surface of the working electrode.^[35]^ The parameter Q, a constant of the constant phase element (CPE), has been used to calculate the pseudo capacitance *C* according to Brug's formula, as reported in the literature.^[36]^ The results show the most significant increase in the charge transfer resistance (R_CT_) with immersion time occurred in the inhibited solution of [DTA]Cou, suggesting that the inhibitor was more effective in blocking the reaction sites.^[37]^ Furthermore, the efficiency extrapolated from the fitting was 88.8 after 24 h, close to the one extrapolated from the Tafel plot (88 %). However, while the R_CT_ of this solution increases with time over 24 h, the efficiencies extrapolated from the fitting after 1 h and 12 h show a decrease in the relative inhibition efficiency over this time. Moreover, while the pseudo capacitance of the double layer decreases for the control over time (which can be related to the formation of corrosion products), it increases for the inhibited solution.

#### Potentiodynamic Polarization (PP)

**Figure 11:**
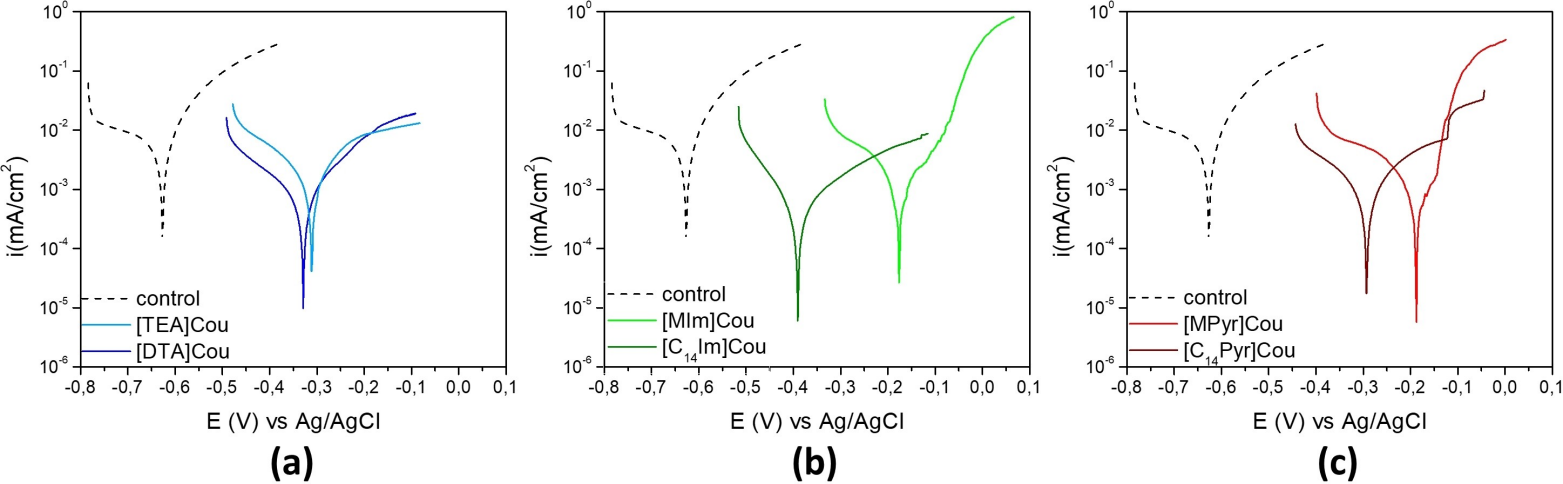
Potentiodynamic Polarization (PP) results of AS1020 mild steel after 24 h in control (0.01 M NaCl; dashed line) and inhibited solutions (0.01 M NaCl + inhibitor 8 mM) containing [TEA]Cou and [DTA]Cou (a), [MIm]Cou and [C_14_Im]Cou (b), [MPyr]Cou and [C14Pyr]Cou (c).

The polarization curves for AS 1020 mild steel after 24 h of immersion in the control (0.01 M NaCl) and inhibited solutions (0.01 M NaCl + inhibitor 8 mM) in are shown in Figure 11, while the corrosion potential (*E_corr_*), corrosion current density (*i_corr_*) and inhibitor efficiency (IE) extrapolated through Tafel plots are reported in Table 2. The inhibitor efficiency, calculated from Equation (1), is a useful tool for comparing the inhibition ability of various inhibitors. However, IE alone does not provide sufficient information about the quality of the inhibitor, as it is affected by the type of corrosion, the stability of the electrochemical system, and presumes linearity in Tafel plots. Therefore, further analysis (such as SEM) is required to corroborate the results obtained for a comprehensive assessment of inhibitor efficiency.

**Table 2:** Corrosion parameters obtained from PP using Tafel extrapolation, in control and inhibited solutions after 24 h of immersion.

Solution	E_corr_ (mV)	Std. Dev.	*i*_corr_ (μA/cm^2^)	Std. Dev.	IE (%)
Control	−630	7	1.51	0.1	‐
[Tea]Cou	−311	8	0.36	0.08	76
[Dta]Cou	−334	12	0.18	0.07	88
[Mpyr]Cou	−187	10	0.29	0.08	81
[C_14_pyr]Cou	−293	4	0.22	0.1	85
[Mim]Cou	−182	14	0.28	0.07	81
[C_14_im]Cou	−396	5	0.12	0.1	92

The addition of the coumarate‐based inhibitors to the 0.01 M NaCl solution resulted in a positive shift of the corrosion potential and a substantial reduction in the corrosion current. From the PP curves reported in Figure 11, it is clear that incorporating a long alkyl chain results in a less pronounced shift of the corrosion potential towards more positive values compared to inhibitors with shorter chains, particularly for[C_14_Im]Cou. Additionally, all the long chain inhibitors demonstrated more effective reduction of the corrosion current compared to their respective short alkyl chain counterparts.

The polarization curves for [TEA]Cou and [DTA]Cou in Figure 11 **(a)**, together with the IE(%) and *i_corr_* reported in Table 2, show that [DTA]Cou achieves a lower corrosion current and greater efficiency compared to [TEA]Cou. Interestingly, [DTA]Cou presents the same slope as [TEA]Cou on the cathodic branch but at a lower position in the anodic branch, [DTA]Cou initially exhibits a lower corrosion current until −0.25 V, where an inflection point occurs. This inflection, resembling a pitting potential, leads to an increase in slope, whereas [TEA]Cou maintains a plateau. Comparing [MIm]Cou and [C_14_Im]Cou (Figure 11 **(b)**), the introduction of a long alkyl chain results in a *E_corr_* of −0.39 V for [C_14_Im]Cou, while [MIm]Cou present a *E_corr_* of −0.18. [C_14_Im]Cou also presents a significant decrease in the anodic branch, especially if compared with [MIm]Cou. From Figure 11 **(b)** and **(c)** is possible to see that [MIm]Cou and [MPyr]Cou show some anodic inhibition, but are unable to supress it effectively, as shown by a pitting‐potential like rapid increase in current close to E_corr._ The PP for the longer alkyl chain inhibitors either do not show this feature or it is further from E_corr._.

The polarization curves of [C_14_Im]Cou and [C_14_Pyr]Cou show that these ILs acted as anodic inhibitors reducing the anodic branch, in correlation with the microscope images that show an almost pristine surface.

[C_14_Im]Cou and [DTA]Cou presented the highest decrease in the corrosion current (0.12 and 0.18 μA/cm^2^) and highest efficiencies, coherently with the results obtained from the immersion specimens and PEIS measurements.

#### Incorporation of Coumarate IL Into an Acrlic UV‐Coating and its Anti‐Corrosion Properties

**Figure 12:**
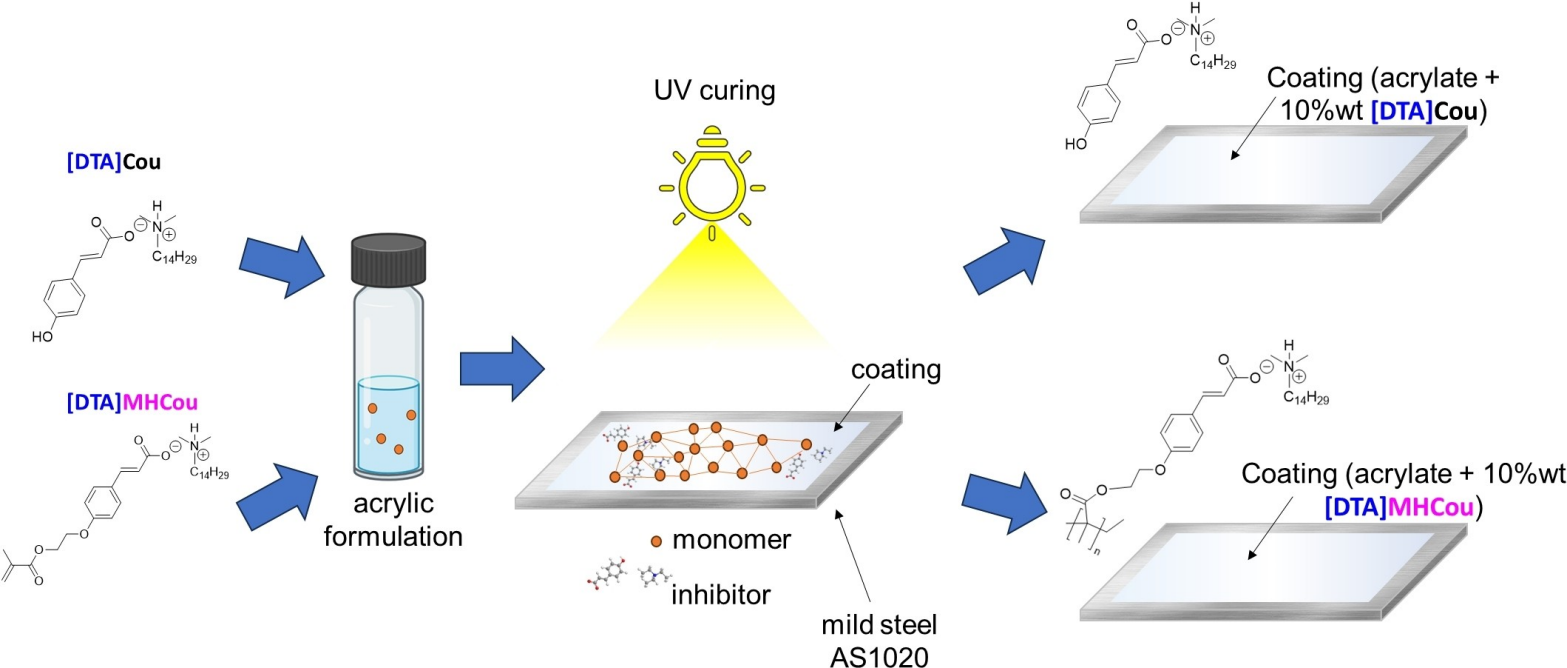
Incorporation of the ILs into a coating as additive([DTA]Cou), and by polymerizing the coumarate anion [DTA]MHCou.

**Figure 13:**
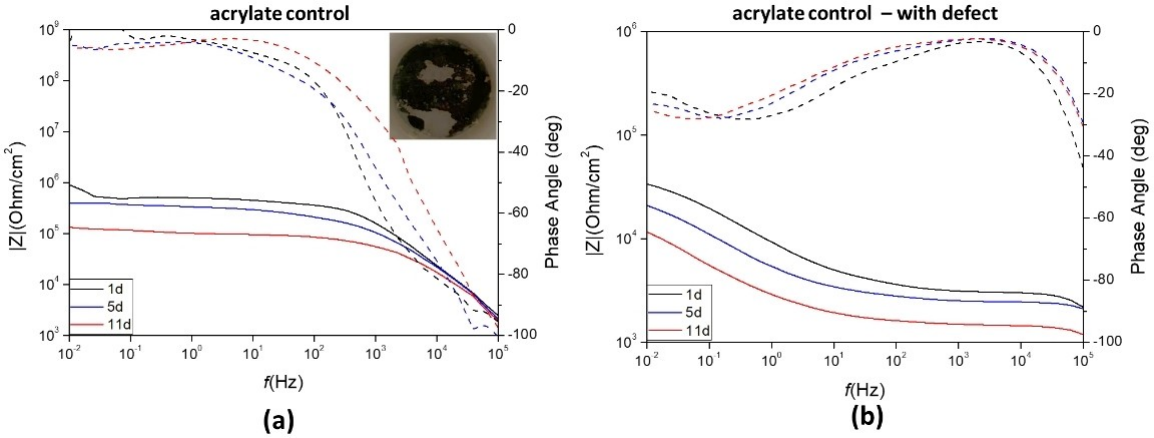
Bode plots of impedance and phase angle (dotted lines) for of AS1020 mild steel coated with acrylic formulation only (control coatings) without (a) and with a defect (b) after 1, 5 and 11 days of immersion. Image of the intact coating after 11 days is also shown.

The inhibitor [DTA]Cou was incorporated into an acrylic UV‐ coating formulation using two different approaches (Figure 12). This IL was chosen due to its good miscibility and compatibility with the acrylic formulation, and for the promising results observed from its analysis when dissolved in solution. . In a first approach [DTA]Cou was directly added to the acrylic formulation in a concentration of 10 wt %. Meanwhile, in a second approach, a similar methacrylic monomer [DTA]MHCou was incorporated also at 10 wt % into the acrylic formulation to investigate the effect of covalently linking the coumarate anion to the polymer coating matrix. Each coating was investigated using PEIS during 11 days of immersion in 0.01 M NaCl solution. The coatings had an average thickness of 60 μm and were investigated intact and with a 1 mm circular defect. Bode plots of the control coating with and without the defect are reported in Figure 13.

#### Incorporation of [DTA]Cou

**Figure 14:**
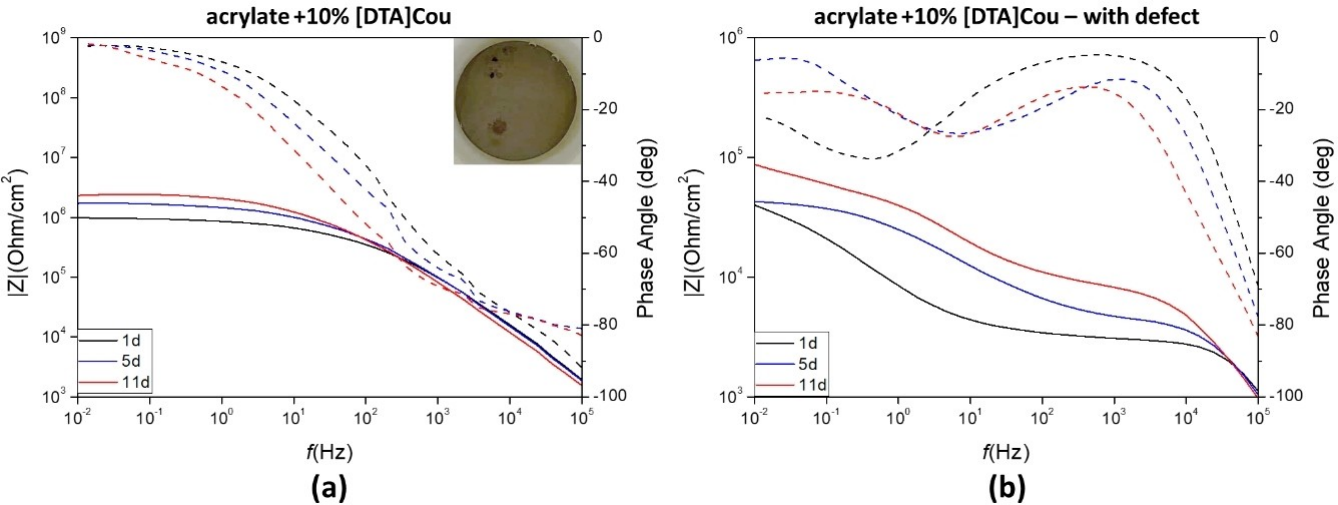
Impedance plot (solid lines) and phase angle plot (dotted lines) of AS1020 mild steel coated with acrylic formulation containing 10 wt % of [DTA]Cou without **(a)** and with **(b)** a defect introduced, exposed to a 0.01 M NaCl solution after 1, 5 and 11 days of immersion. Image of the intact coating after 11 days is also shown.

In Figure 14 the Bode plots for pristine and defect containing coatings during the 11 days of exposure to a 0.01 M NaCl solution are reported.

At low frequencies the control coating(Figure 13), exhibits an impedance value of 50×10^4^ Ohm/cm^2^ and a phase angle of −3,3° after one day, which decreased to 10×10^4^ Ohm/cm^2^ and −18° respectively after 11 days of exposure, indicating deterioration of the coating during time. The plots of impedance values at 0.01 Hz vs immersion time are reported in Figure S15. After 11 days the coating was still adhering on the surface but showed evident signs of damage and corrosion underneath.

After one day the specimen coated with the acrylic formulation doped with 10 wt % [DTA]Cou displays an impedance value of approximately 10^5^ Ohm/cm^2^ in the low frequency range (10^−2^–10^0^ Hz). In this case, an increase in impedance over the 11 days can be observed.The increase in impedance with time could indicate that the inhibitor is present at the surface, or is able to leach out of the coating and form a second protective layer on the sites where electrolyte penetrates and corrosion would initiate.^[19]^ The impedance plots of the control coating reach a plateau at approximately 10^3^ Hz, while the impedance for the [DTA]Cou coated specimen continue to increase, plateauing at a lower frequency range (10^1^–10^2^ Hz).

When a defect is applied, the impedance of the control was 3.4 x10^4^ Ohm/cm^2^ after 1 day and constantly decreased over 11 days to 1×10^4^ Ohm/cm^2^. With 10 wt % of the inhibitor added into the coating, the impedance after 1 day was 4×10^4^ Ohm/cm^2^ and increased to 8.8×10^4^ Ohm/cm^2^ after 11 days. Similar to the defect‐free coating, the impedance increased with immersion time, thus showing active corrosion protection in both cases.

#### Incorporation of [DTA]MHCou

**Figure 15:**
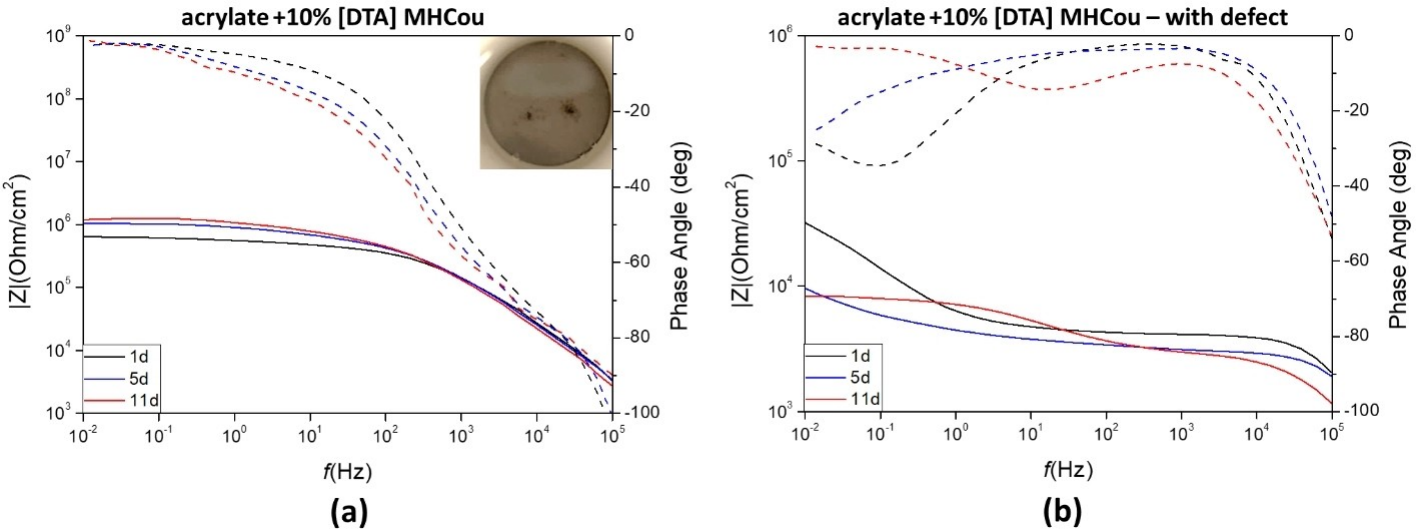
Impedance plot (solid lines) and phase angle plot (dotted lines) of coated AS1020 mild steel containing 10 wt % of [DTA]MHCou without **(a)** and with defect **(b)** exposed to a 0.01 M NaCl solution after 1, 5 and 11 days of immersion. Image of the intact coating after 11 days is also shown.

Figure 15 shows the recorded impedance spectra and phase angle plots of steel panels coated with the diacrylate formulation containing 10 wt % of [DTA]MHCou without **(a)** and with **(b)** a defect, after 1, 5 11 days of exposure to a 0.01 M NaCl solution. After 11 days, the impedance modulus of the sample coated with the formulation containing 10 wt % of the monomer [DTA]MHCou increased in the middle (10–10^3^ Hz) and low frequencies range (10^–1^–10^−2^ Hz) with respect to the first day, reaching similar levels to the [DTA]Cou. If compared to the plot in Figure 14 **(a)**, when the inhibitor is “free” and not covalently linked to the coating, it is possible to see that the values of impedance in the low frequency ranges (10^–3^–10^0^ Hz) are quite similar, but they present a different time constant, which appears higher in the case of [DTA]Cou. The difference is more pronounced in the presence of a defect, since [DTA]MHCou in Figure 15 **(b)** presents an impedance that strongly decreases from 1 to 5 days of exposure. After 11 days, the different slope of the plot can be caused by the amount of corrosion products on the defect site. In the case of a defect present in the coating, while [DTA]Cou showed an increase in the impedance as the exposure time increases, [DTA]MhCou presents a decrease in the impedance over time at low frequencies. These results suggest that when the coating is intact the presence of the inhibitor at the interface is important to improve performance, as the results for the ‘free’ and bonded inhibitors are similar, however when a defect is present the ability of the inhibitor to leach into solution and react at the exposed metal is important. Since in the case of [DTA]MhCou the linking of the anion to the matrix does not allow the inhibitor to exhibit its inhibiting properties on the defect site, the performance of the coating in this case strongly decreases.

## Conclusions

In this work, six coumarate based ILs were evaluated as corrosion inhibitors for AS1020 mild steel in a 0.01 M NaCl aqueous solution. The results obtained from the tests in solution showed that an increase in carbon chain length on the cation results in an enhancement of the inhibition efficiency and mitigation of pitting corrosion. In particular, [C_14_Im]Cou and [DTA]Cou showed the highest efficiencies (91.9 % and 88 %) and lowest corrosion currents. From the SEM micrographs and EDS analysis, the steel surface exposed to the inhibited solution of [DTA]Cou appeared almost unaffected and with few small deposits, while the polishing marks remained clearly visible. On the other hand, the inhibitors presenting a short alkyl chain demonstrated a poor inhibition effect with clear signs of corrosion after the 24 h of immersion.

One of these inhibitors, [DTA]Cou, was incorporated in an acrylic coating using two different methods. In the first approach, [DTA]Cou was added by simple mixing it into the formulation. In the second case, a methacrylate moiety was added to the anion ([DTA]MHCou) to bond it with the polymer matrix. When 10 wt % of the [DTA]Cou was incorporated into the acrylic coating enhanced the corrosion protection of these UV‐cured coatings for mild steel compared to a control was evident. After 1 day an increase in impedance of almost one order of magnitude occurred, and this difference became more evident with time. Furthermore, the bonding of the coumarate to the polymer matrix by photopolymerization of the methacrylate moiety showed that the addition of 10 wt % of the monomer [DTA]MHCou resulted in an impedance of 12×10^5^ Ohm/cm^2^ after 11 days, which was higher than the control (9×10^5^ Ohm/cm^2^) but lower then [DTA]Cou,( 8.8×10^4^ Ohm/cm^2^). In the presence of a defect the [DTA]Cou containing coating was still able to show active inhibition, with an increase in impedance over time, almost reaching 1×10^5^ Ohm/cm^2^ after 11 days, however the control and [DTA]MHCou performed similarly, decreasing to 1×10^4^ Ohm/cm^2^.These results not only confirm the key role of the coumarate anion in the inhibition process of corrosion but suggest that the coumarate is fundamental to improve the barrier properties. Although integrating an inhibiting molecule into the acrylic polymer improved the anticorrosion properties of the intact UV cured coatings, the inhibition of corrosion in the presence of an introduced defect was more pronounced with [DTA]Cou compared to coumarate linked to the polymer matrix. This highlights the critical role of the coumarate anion in corrosion inhibition.

In conclusion, coumarate‐based ILs inhibitors demonstrated excellent performance, particularly with the introduction of a long alkyl chain in the cation. The incorporation of [DTA]Cou as an additive into the coating and [DTA]MhCou by polymerizing the anion showed promising results but, differently from similar systems,[^22^, ^38^] these seem to be the only ones that show an increase in impedance with time, and the longest tested. Compared to previous works, the investigated systems present better performance that allowed the coatings to be analyzed for a longer time.

## Supporting Information Summary

Supporting information is available online.

## Conflict of Interests

The authors declare no conflict of interest.

1

## Data Availability

The data that support the findings of this study are available from the corresponding author upon reasonable request.
